# Thermostatic regulation of blood samples in blood gas analysers: results and improved inethod applied to 11 different models

**DOI:** 10.1155/S1463924682000340

**Published:** 1982

**Authors:** B. Dingeon, H. Bernon, A. Roullet, C. Collombel

**Affiliations:** 1 Laboratoire de Biochimie Centre Hospitalier Chambery F 73011 France; 2 Laboratoire de Biochimie Hôpital Jules Courmant Pierre Benite F 69310 France; 3 Service de Biochimie C Hôpital Edouard-Herriot 5 Place d'Arsonval Lyons F 69003 France


Journal of Automatic Chemistry, Volume 4, Number 3 (July–September 1982), pages 135–138

### Thermostatic regulation of blood samples in blood gas analysers: results and improved method applied to 11 different models

**B. Dingeon**

*Laboratoire de Biochimie, Centre Hospitalier, F 73011 Chambery, France*

**H. Bernon, A. Roullet**

*Laboratoire de Biochimie, Hôpital Jules Courmant, F 69310 Pierre Benite, France*

**and C. Collombel**

*Service de Biochimie C, Hôpital Edouard-Herriot, 5 Place d'Arsonval, F 69003 Lyons, France*

### Introduction

As part of a general assessment of blood gas analysers [1] the thermostatic regulation of blood samples in the machines' sample chambers was studied with the aid of a thermal probe placed on one of the electrodes. The experiment was necessary because the analysers' readings assume a temperature of 37°C; in principle the instruments should maintain this temperature to within 0·1°C.

The influence of temperature varies with each parameter being measured (for example pH, percentage oxygen [$pO_2$] and percentage carbon dioxide [$pCO_2$]) and according to the nature of the solution being analysed [2]. For protein solutions, and especially for blood samples, the average variations are: for pH, 0·011 units per degree; for $pCO_2$, 4%/°C, and for $pO_2$, 7%/°C. In addition, cooling of the electrodes modifies these variations: for $pO_2$ this may vary by as much as 10%/1°C; for $pCO_2$ the effects of temperature are in part compensatory, reducing the variations to −2% error/1°C [3, 4].

Aqueous buffers are less sensitive to temperature; they are not therefore suitable for thermostatic control [5].

The measuring electrode in each instrument was modified. The sensing part of the $pO_2$ or the $pCO_2$ was replaced by the heat-sensitive end of a thermocouple.

The aim of this investigation was to examine the temperature of the sample itself within the sample chamber, without disturbing the normal flushing, calibrating and measuring processes which are very elaborate in some 'automatic' instruments.

### Materials and methods

### *pH blood gas analyser*

A wide range of instruments, 11 different models, produced by five international manufacturers (Corning, BP 36, 11 Chemin de Ronde, F 78110 Le Vesinet, France; Technicon, Route Nationale No. 1, F 95330 Domont, France; Instrument Laboratory—Delhomme, 32 avenue St Mandé, F 75562 Paris Cedex 12; Radiometer—Jarre Jacquin, 30 rue St Augustin, F 75002 Paris; and AVL, Place de l'Europe, F 94019 Creteil, France) were tested. See table 1 for a list of the machines and the results obtained. For each model (except one) two instruments were tested to ensure the validity of the results.

### *Thermocouple*

The thermocouple used was a Constanant which has a diameter of 1 mm, a very short response delay of 3 s, and a theoretical sensitivity of 4 MV/°C. Thus 0·01°C can be estimated ensuring a precision 0·1°C.

The thermocouple was fixed on each instrument's jacket in place of the $pCO_2$ electrode. The thermocouple was passed through a silicon rubber plug (cut to size), and pushed firmly to the bottom of the jacket in order to keep the system watertight. A large diameter trocar (3 mm) was used to pass the thermocouple through this stopper. Once the trocar is removed, the thermocouple remains held in place by the elasticity of the stopper (see figures 1 and 2).

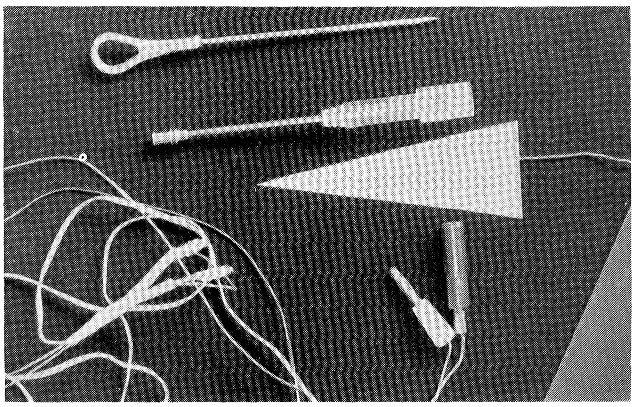

*Figure 1. Parts required for assembling the thermocouple.*

Each electrode assembly fitted with the thermocouple was then calibrated in a water bath with a circulating system, at three or four different temperatures ranging from 36°C to 38°C.

The same high-precision thermometer (manufactured by Walter-Kessler, Z.A. Courtaboeuf, F 91403 Orsay, France), graduated to 0·02°C, was used throughout the investigation. A calibration curve was drawn for each instrument (figure 3). Zero was obtained by plunging the two ends of the thermocouple into a thermos flask filled with distilled water and ice. The iced water served throughout each experiment as a fixed temperature of reference.

0142 0453/82/0403 0135 $05·00 © 1982 Taylor & Francis Ltd

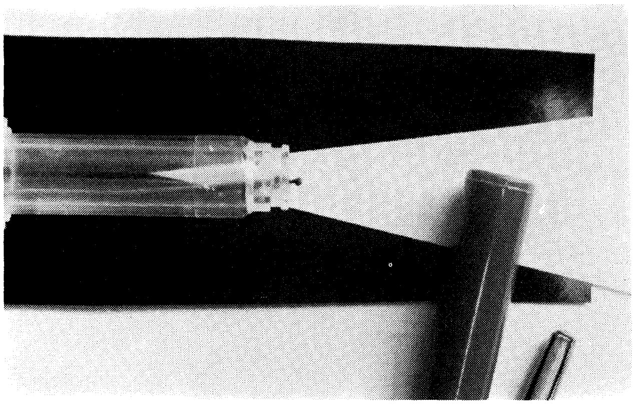

*Figure 2. Thermocouple fixed on an instrument's jacket. Note the fineness of the sensitive part of the thermocouple.*

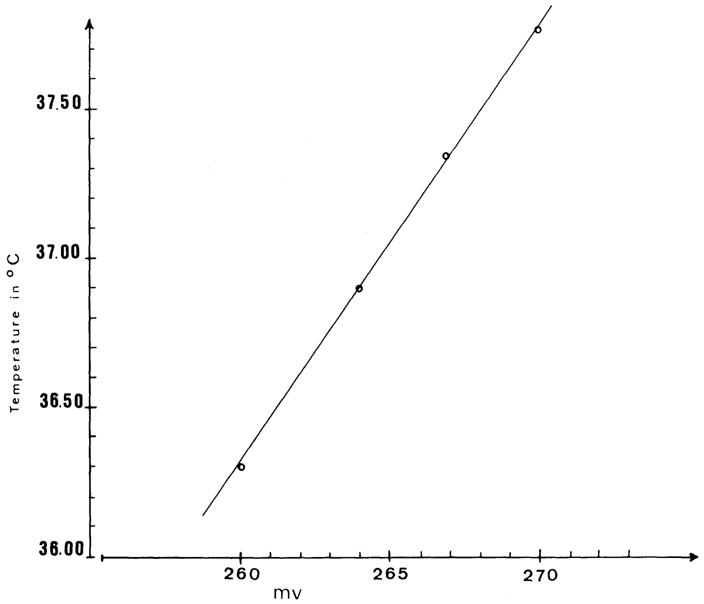

*Figure 3. Calibration of the thermocouple fixed on the instruments' jackets.*

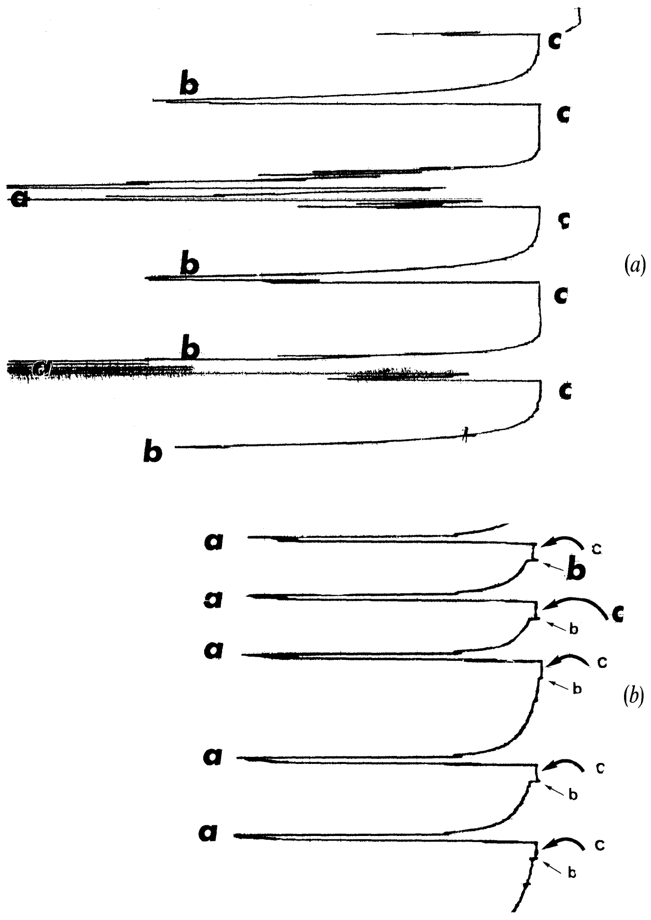

*Figure 4. Examples of recorded temperatures (1 square = approximately 0·13°C). Figure 4(a) is from an instrument without a pre-heater (speed of paper was 10 mm/min.) and figure 4(b) is from an instrument with a pre-heater (speed of paper was 5 mm/min.); a = rinsing, b = entry of sample, c = final temperature.*

### *Recorder*

The recorder used was a Kipp and Zonnen micrograph BDN°5. Temperatures were recorded during the instrument calibration with gas; during three successive measurements of one blood sample previously maintained at 25°C, and during the three successive measurements of the same blood sample previously maintained at +4°C. In each case the manufacturer's operating instructions were followed and the timing programmes of the instruments were taken into account. The thermocouple system indicates the temperature variations in the sample chamber when the gas is passed through, although the values obtained, due to the gas, are not necessarily exact. The continuous recordings of temperatures from within the sample chamber are illustrated in figure 4.

### Results

The results of the study are shown in table 1; the instruments are listed there according to their degree of automation. The notes below relate to the table.

(*a*) In this instrument the galvanometer indicating temperature reverts to 37°C 30 s before complete thermal equilibrium of the sample.

(*b*) The instrument confirms its results after about 45 s although the thermal balance of the sample is reached after 2 min. 10 s at 37·12°C.

(*c*) After rinsing with the non-thermostated solutions the temperature of the measuring chamber is slow in returning to its exact level (3 min.). In addition it was noted with this instrument that too great a speed of gas flow significantly lowered the temperature of the chamber (the calibration gases do not pass through the pre-heater). The flow rates advised by the manufacturer should be followed.

(*d*) See note (*b*).

(*e*) This instrument was adjusted by the manufacturer just before testing: this could explain the quality of the results.

(*f*) The thermal equilibrium of the sample is reached after about 2 min. 30 s at 36·85°C. The instrument thus confirms its results before thermal equilibration is reached, hence the difference in values observed.

(*g*) After reassembly of the instrument the test was not carried out until the temperature warning light was extinguished.

*Table 1. Temperature of blood samples in the measuring chamber.*

| Instrument | | Blood stored at 25°C — Values of the three readings in °C | | | Blood stored at +4°C — Values of the three readings in °C | | | Isothermic time lag | Remarks |
|---|---|---|---|---|---|---|---|---|---|
| Corning 161 | (1) | 36·94 | 36·94 | 36·94 | 36·94 | 36·90 | 36·90 | 1 min. 20 s | *a* |
| | (2) | 36·85 | 36·83 | 36·87 | 36·81 | 36·78 | 36·80 | 1 min. 30 s | *a* |
| BMS $MK_2$ | (1) | 36·88 | 36·76 | 36·82 | 36·80 | 36·80 | 36·86 | 3 min. | |
| Radiometer | (2) | 36·62 | 36·62 | 36·62 | 36·62 | 36·62 | 36·62 | 3 min. | |
| IL 213 | | 36·90 | 36·85 | 36·85 | 36·86 | 36·90 | 36·90 | 3 min. | |
| Corning 166 | (1) | 37·00 | 36·85 | 36·85 | 36·85 | 36·85 | 36·76 | 2 min. 20 s | *a* |
| | (2) | 37·02 | 37·04 | 37·02 | 37·05 | 37·05 | 37·05 | 2 min. 20 s | *a* |
| AVL 938 | | 36·60 | 35·0 | — | 36·66 | 35·02 | — | 2 min. 10 s | *b* |
| IL 413 | (1) | 36·62 | 36·68 | 36·68 | 36·68 | 36·68 | 36·68 | 5 s | *c* |
| | (2) | 36·72 | 36·70 | 36·72 | 36·50 | 36·56 | 36·56 | 5 s | *c* |
| IL 613 | (1) | 36·75 | 36·60 | 36·60 | 36·65 | 36·60 | 36·60 | 5 s | *c* |
| | (2) | 36·69 | 36·63 | 36·60 | 36·64 | 36·60 | 36·70 | 5 s | *c* |
| AVL 940 | (1) | 36·46 | 36·53 | 36·60 | 36·46 | 36·50 | 36·60 | 1 min. 30 s | *d* |
| | (2) | (37·05) | (37·05) | (36·94) | (37·05) | (37·05) | (36·96) | 1 min. 30 s | *e* |
| Corning 175 | (1) | 36·98 | 36·98 | 37·04 | 37·04 | 37·16 | 37·10 | 50 s | *a* |
| | (2) | 37·10 | 37·12 | 37·12 | 37·12 | 37·10 | 37·12 | 1 min. | *a* |
| Technicon BG II | (1) | 36·60 | 36·68 | 36·68 | 36·66 | 36·48 | 36·75 | 2 min. 30 s | *f* |
| | (2) | 36·44 | 36·28 | 36·55 | 36·50 | 36·48 | 36·56 | 2 min. 30 s | *f* |
| $ABL_2$ | (1) | 36·24 | 36·30 | 36·30 | 36·30 | 36·30 | 36·30 | 10 s | *g* |
| Radiometer | (2) | 36·48 | 36·46 | 36·46 | 36·46 | 36·46 | 36·44 | 10 s | *g* |

The table shows temperatures of samples when the operator, or automatic instrument, confirms the result, but not necessarily the temperature of the sample at calorific balance.

### *Accuracy*

The lowest temperature measured was 36·24°C and the highest 37·16°C. One company, Corning, stands out as ensuring a high quality of sample thermoregulation on the whole range of their instruments, irrespective of the level of automation. All other models show greater or lesser inexactitude, erring always by default.

### *Repeatability*

Table 1 demonstrates that, on the whole, the repeatability of temperature is very good for a given instrument.

### *Influence of sample temperature*

It can equally be seen that the initial temperature of the sample does not affect its final temperature in the sample chamber. This holds good whether the instrument has a pre-heater or not. This fact means that if measuring has to be deferred (due to work-loads in the laboratory or any other reason) the blood samples can be refrigerated (at +4°C).

### Comments

It was noticed in the course of these investigations that too high a flow rate of gas in the sample chamber during calibration cooled the chamber (from between 1 and 1·5°C) and this invalidates calibration values.

In some instruments (AVL particularly), the copious flow of cleaning fluids which are not thermostatically controlled leads to significant cooling in the sample chamber, which then takes about 5 min. to regain its correct temperature this reduces the analytical throughput of samples.

For instruments which have no fixed time input the steady state of sample temperature may be reached if the response time of electrodes is slow. On the other hand, when the response time is fast, the results are read before the steady state.

When pre-heaters are provided with the instrument the blood sample enters the sample chamber at a temperature very near to the steady state.

### Conclusion

Twenty instruments—11 models from five manufacturers—were tested; it was concluded that only one company offers instruments which conform technically with the documentation supplied.

The other companies' instruments fail in accuracy but good repeatability of sample thermostatization is obtained; therefore it should be possible to overcome this problem by correctly adjusting the instruments.

### Acknowledgements

The authors wish to thank the following for their help: Mme F. Volter, Mme Leviel, Mrs Fineti, Mrs Lauriat, Pr. Meunier, Pr. Teisseire, Pr. Sachs (Paris) and Mrs Feuillu (Rennes), Pr. Cabanac (Lyon), Pr. Valdiguie (Toulouse), Pr. Vigneron (Nancy), and the following companies AVL (France), Corning (France), Technicon (France).

This work was sponsored by La Société Française de Biologie Clinique and by the Centre d'Etude des Réactifs et Matériels de Biologie Médicale.

### References

1. VALDIGUIE, P. and COLLOMBEL, C. *et al.*, *Evaluation of Blood Gas Analysers* (C. H. U. Rangueil, Toulouse, 1979).
2. WEISBERG, H. F., *Tri Slide TM Calculator for HENDERSON-HASSELBACH Equation and CO2 RREC°-t-$O_2$-SLIDE$^{TM}$ for temperature corrections* (National Bureau of Standards, Washington, D.C., 1977), pp. 91–101.
3. BAINTON, C. R. and SEVERINGHAUS, I. W., *Anesthesiology*, **33** (1970), 548–550.
4. FINETTI, P., *Bulletin de Physiopathologie respiratoire*, **4** (1968), 509–536.
5. MANNING, J. P., SASAKI, D. N. and WERTLAKE, P. T., *Clinical Chemistry*, **20** (1974), 1226–1228.

### Call for papers

The 14th Annual Symposium on the Analytical Chemistry of Pollutants and the 3rd International Congress on Analytical Techniques in Environmental Chemistry will be held from 22–24 November 1984 at the Palacio de Congresos in Barcelona, Spain. After the two meetings several workshops on specific topics will be held—these include a workshop on ion chromatography and another on the chemistry and analysis of hydrocarbons in the environment. Those wishing to submit a paper or poster should send the organizers a 200-word abstract by 15 December 1983. Papers will be refereed and the official language is to be English.

A commercial exhibition—Expoquimia 84—will run concurrently with the meetings.

*Details from 14th Annual Symposium-3rd International Congress-Expoquimia, Avda Reina M.ª Christina, Barcelona 4, Spain.*

### Notes for Authors

*Journal of Automatic Chemistry* covers all aspects of automation and mechanization in analytical, clinical and industrial environments. The *Journal* publishes original research papers; short communications on innovations, techniques and instrumentation, or current research in progress; reports on recent commercial developments; and meeting reports, book reviews and information on forthcoming events. All research papers are refereed.

### Manuscripts

Two copies of articles should be submitted to the Editor. All articles should be typed in double spacing with ample margins, on one side of the paper only. The following items should be sent: (1) a title-page including a brief and informative title, avoiding the word 'new' and its synonyms; a full list of authors with their affiliations and full addresses; (2) an abstract of about 250 words—this should succinctly describe the scope of the contribution and highlight significant findings or innovations; it should be written in a style which can easily be translated into French and German; (3) the main text with sections and subsections numbered; (4) appendices (if any); (5) references; (6) tables, each table on a separate sheet and accompanied by a caption; (7) illustrations (diagrams, drawings and photographs) numbered in a single sequence from 1 upwards and with the author's name on the back of every illustration; captions to illustrations should be typed on a separate sheet.

### References

References should be indicated in the text by numbers following the author's name, i.e. Skeggs [6]. In the reference section they should be arranged thus:

to a journal

MANKA, D. P., *Journal of Automatic Chemistry*, **3** (1981), 119.

to a book

MALMSTADT, H. V., in *Topics in Automatic Chemistry*, Ed. Stockwell, P. B. and Foreman, J. K. (Horwood, Chichester, 1978), p. 68.

### Illustrations

Line diagrams are preferred to photographs. Original copies of diagrams and drawings should be supplied, and should be drawn to be suitable for reduction to the page or column width of the *Journal*, i.e. to 85 mm or 179 mm, with special attention to lettering size. Photographs may be sent as glossy prints or as negatives.

### Proofs and offprints

The principal or corresponding author will be sent galley proofs for checking and will receive 50 offprints free of charge. Additional offprints may be ordered on a form which accompanies the proofs.

*Manuscripts should be sent to the Editor: Dr Peter B. Stockwell, Plasma-Therm Ltd, Unit 3, 2/3 Kangley Bridge Road, Lower Sydenham, London SE26 5AW.*

